# Prehospital point-of-care emergency ultrasound: a cohort study

**DOI:** 10.1186/s13049-018-0519-9

**Published:** 2018-06-18

**Authors:** Maximilian Scharonow, Christian Weilbach

**Affiliations:** Department of Anaesthesia, Intensive Care, Emergency Medicine and Pain Therapy, St.Josefs-Hospital Cloppenburg, Krankenhausstrasse 13, 49661 Cloppenburg, Germany

**Keywords:** Prehospital, Ultrasound, Trauma, Dyspnoea, Resuscitation, Heart failure, Pulmonary disease, Sensitivity, Quality

## Abstract

**Background:**

In the prehospital situation, the diagnostic armamentarium available to the rescue physician is limited. Emergency ultrasound has proven to be a useful diagnostic tool, providing crucial information for the management of critically ill and injured patients. The proportion of performed ultrasound scans in all patients attended to by the rescue service team, the quality of the findings and the ultrasound-related changes in management approach and patient transport were evaluated.

**Methods:**

In this prospective 18-month observational study, we documented all missions performed by rescue physicians with special training in emergency ultrasound (expert standard). These data were than analysed with regard to the ultrasound examinations. The ultrasound protocols used comprised Focussed Assessment with Sonography for Trauma (FAST), Prehospital Lung Ultrasound (PLUS) and Focused Echocardiography in Emergency Life support (FEEL). The quality of prehospital examinations was assessed by comparing the findings and diagnoses at the emergency site with those established in hospital. The changes in patient management and transport were documented using a standardized protocol.

**Results:**

A total of 99 (18.1%) emergency ultrasound examinations were performed during 546 callouts. The most common indications for prehospital emergency ultrasound were dyspnoea (*n* = 38; 38.4%), during cardiac arrest (*n* = 17/17.2%), fall (*n* = 12/12.1%) and high-speed trauma (*n* = 11/11.1%). The combinations of ultrasound examination protocols in the trauma group (*n* = 31; 31.3%) were: 1. FAST+FEEL+PLUS (*n* = 17; 54.8%). 2. FAST+PLUS (*n* = 11; 35.5%) 3. FAST alone (n = 3; 9.7%). In the non-trauma group (*n* = 68; 68.7%), the following combinations were used: 1. FEEL+PLUS (*n* = 36; 52.9%), 2. FEEL alone (*n* = 21/30.9%). 3. PLUS alone (n = 6/8.8%) 4. FAST alone (n = 2; 2.9%) 5. FAST+FEEL+PLUS (n = 2; 2.9%). 6. FAST+FEEL (*n* = 1/1.5%). The emergency ultrasound findings impaired left ventricular contractile function (sensitivity 89.4%), right ventricular stress (85.7%), lung interstitial syndrome (100%), ruling out pneumothorax (specificity 100%), ruling out intraabdominal fluid (97,1%) were verified at the receiving hospital using ultrasonography, CT scan or x-rays; the prehospital diagnosis was confirmed in 90.8% of cases, the difference between the prehospital and in-hospital findings were not significant(*p*-values from *p* = 0.688 to p = 0,99). Ultrasound-related changes in patient management occurred in 49.5% of patients; in 33.3%, these were transported-related.

**Conclusions:**

Emergency ultrasound was as often used in the prehospital situation as it is in hospital. The ultrasound findings correlated well with in-hospital diagnostic results. Significant pathology changed patient-management, without prolonging the mission time.

## Background

At the scene of an emergency, the diagnostic armamentarium available to the emergency physician, in addition to clinical examination and history, is limited to “basic monitoring”, including ECG, pulse oximetry, cuff-based blood pressure measurements, and, following intubation, measurements of CO2 in expired air by means of capnometry. However, some critical diagnostic questions cannot be addressed using these tools [1–6].

In an emergency situation, ultrasonography can provide guiding insights into a patient’s condition or injury pattern [1, 3–7] and is considered to be a highest-priority technological tool that deserves evaluation [8].

In trauma patients, guiding ultrasound findings with a direct impact on acute patient management are intraabdominal or intrathoracic free fluid, [9] and pneumothorax [4, 10].

In critically ill patients with e.g. hypotension, ultrasonography can help to identify the underlying condition, such as impaired myocardial contractile function, hypovolaemia, pericardial effusion, pulmonary embolism, among other, so that further treatment can be initiated with more confidence [1], as already been shown for the treatment in the hospital [11]. This is of special importance since some treatment approaches may lead to opposite effects. This also applies to dyspnoea as the principal symptom; in this case, ultrasonography can differentiate between lung interstitial syndrome and lung consolidation as the underlying condition.

For example, if in a patient with critical hypotension significant intraabdominal free fluid is ultrasonographically detected after trauma, the treatment would be different to the same patient without free fluid, but with signs of congestive heart failure (impaired left ventricular contractile function, lung interstitial syndrome).

In contrast to the now widespread adoption of ultrasound scanners in departments of anaesthesiology and intensive care units, mobile ultrasound systems are less commonly used in prehospital emergency medicine [9]. This may be due to the fact that these mobile scanners are not part of the standard equipment for rescue service vehicles covered by public payers in Germany, so funding has to be obtained from other sources (district administration, donations etc.).

### Research question

The aim of this study on prehospital emergency ultrasound is to evaluate the range of valuable prehospital situations in which ultrasound was used, the quality of ultrasound findings and other factors influencing the rescue service mission and patient transport, if the same indication criteria as those applied to in-hospital (admission) examinations are used.

## Methods

### Study design and setting

Designed as an observational study, we conducted a research project at a rural rescue service base in which we documented and analysed with regard to emergency ultrasound all rescue service missions during an 18-month period which were performed by rescue physicians with special training in emergency ultrasound who also received continuing education in emergency ultrasound and supervision after each mission.

### Participants

Eligible for performing these ultrasound examinations were rescue physicians who had regularly worked as rescue physicians for a period of at least 5 years and used ultrasound in the prehospital situation after completion of the relevant ultrasound courses (FAST, FEEL, AFS modules). In addition, these physicians experienced in emergency medicine participated in weekly ultrasound training sessions in the Department of Anaesthesiology and regularly performed specialist-grade ultrasound examinations as part of their daily clinical routine (intensive care unit, anaesthesia).

### Variables

The introduction of prehospital emergency ultrasound at the studied rescue service base and the preceding and ongoing training provided to rescue physicians were supported and described by a prospective study [1, 2, 9, 10, 12]. The questionnaires and protocols developed for the initial study were used further in this study for documentation and recorded, apart from patient-related information, the leading symptoms or emergency situations that triggered FAST, FEEL and PLUS examinations, the ultrasound examination time, the findings, and changes in patient management and transport organisation. Prior to the start of this study, situations leading to prehospital emergency ultrasound were defined based on the results of previous studies [1, 6]. The examinations (FAST, PLUS, FEEL) were triggered by both clinical symptoms (dyspnoea, cardiovascular arrest, abdominal pain, chest pain, among others) and the mechanism of injury (high-speed trauma, fall from a height > 2 m, among others).

### Data sources/measurement

Biometric and mission-related data (date, time, duration of mission, diagnosis, speciality, severity of disease/injury, etc.) from a standard emergency protocol filled out by a rescue physician (DIVIDOK Version EPRO-01030801) as well as the documentation of the FAST, FEEL and PLUS examinations (modified protocol from the previous study [6]) were collected over the entire study period.

The ultrasound scans at the emergency site were performed using a mobile ultrasound scanner (Sonosite, MicroMaxx / sector array transducer P17/5–1 MHz).

Ultrasound examination times were measured by paramedics, using a stopwatch; the measurement started (with the scanner ready to be used) at the point in time the rescue physician took the transducer and ended at the moment the rescue physician handed the transducer back to the paramedic. The time spent on ultrasound examination during resuscitations was not included in the analysis. In this situation, the prepared ultrasound scanner was used for a maximum of 10 s each time, according to the resuscitation protocol described by Breitkreutz et al. [12], integrated in the resuscitation algorithm of the ERC guideline. In the resuscitation situation, the term “FEEL” is used as a substitute only, as to date no definition of resuscitation-associated ultrasonography has been published.

To verify the quality of the prehospital ultrasound findings and diagnoses, these were compared with the in-hospital findings obtained in the receiving hospitals. For this end, the anonymized hospital discharge reports received by the rescue physicians were analysed by a study nurse, who was not aware of the prehospital examination results. Following parameters were investigated: left ventricular contractile function, lung interstitial syndrome, ruling out intraabdominal fluid, ruling out pneumothorax, and right ventricular stress.

In addition, data with regard to changes in patient management and transport were analysed. Apart from changes in the transport destination, the transport priority and monitoring requirements (accompanied by rescue physician or paramedic) were documented.

### Statistical methods

Data were analysed using the SPSS software package after consultation by the Centre for Biometry, Medical Informatics and Medical Technology at the Hannover Medical School. The statistical tests performed included testing for normality (Kolmogorov-Smirnov^a^, Shapiro-Wilk), the Mann-Whitney U test, the Levene test, and the McNemar test for repeated categorical data, among others.

### Ethics committee approval

After receipt of the approval of the Ethics Committee of the Hannover Medical School (MHH) (No. 2896/2015) and the Ethics Subcommittee of the Medical Association of Lower Saxony, the study was conducted. The analysis of the anonymized mission-related data and the hospital discharge reports received after the rescue physician mission was exclusively performed by the rescue physicians involved.

## Results

During the study period of 18 months, prehospital emergency ultrasound scans were performed in 99 (18.1%) of the 546 patients treated by rescue physicians, either at the emergency site or during patient transport.

Significant differences were found among the biometric data with regard to the parameters listed in Table 1.

**Table 1:** Biometric data

	n (%)	Age (years)	BMI	HR Initially / at handover	BP Initially / at handover	SpO2 Initially / at handover
Total pts.	*n* = 546	57,8 (σ 25,6)				
♂	*n* = 313 (57.3%)	55.0 (σ 24.6)				
	(313vs.233: *p* = 0.001)	(55.0 vs. 61.4: *p* = 0.004)				
♀	*n* = 233 (42.7%)	61.4 (σ 26.5)				
US pts.	*n* = 99 (18.1%)	63.4 (σ 23.7)				
♂ US pts	*n* = 59 (18.8%)	60.1 (σ 22.8)				
	Tr 19 (61.3%)					
	nTr 40 (58.8%)					
♀ US pts	*n* = 40 (17.2%)	68.2 (σ 24.2)				
	Tr 12 (38.7%)					
	nTr 28 (41.2%)					
Trauma	US n = 31 (31.3%)	40.7	24.6	95.0 / 85.6	140.8 / 138.8	97.7 / 98.1 (97.7 vs. 83.1 *p* < 0.001)
		(40.7 vs.73.7: *p* < 0.001)	(24.6 vs. 28.1: *p* = 0.008)	(85.6 vs. 94.4: *p* = 0.046)		(98.1 vs. 94.4: *p* = 0.013)
Non-trauma	US n = 68 (68.7%)	73.7	28.1	87.8 / 94.4	142.2 / 133.5	83.1 / 94.9

*♂* male, *♀* female, *BMI* body mass index, *HR* heart rate, *BP* blood pressure, *US* ultrasound, *pts*. patients, *Tr* Trauma, *nTr* non-trauma, *σ* standard deviation

More than half of the patients assessed with emergency ultrasound (*n* = 99) were medical cases (*n* = 68/68,7%) and about one quarter were trauma surgery cases (*n* = 31/31,3%).

Most patients (based on all 546 missions) were classified as having a moderate to severe but not life-threatening disorders (NACA III), followed by NACA IV and NACA V. Each of the categories NACA I, II, VI and VII accounted for less than 7% of patients (Table 2).

**Table 2:** Admission unit and speciality, NACA, mission time

		Patient admission	NACA	Mission time
Total pts. (n = 546)		Admission unit:	∅ 3.7	34:12 min (σ 15:46)
		CED (*n* = 383/70.3%)	3.7 vs. 4.5: *P* < 0.001 (σ 1.3 / CI 3.55–3.78)	
		ICU (n = 54/9.9%)	NACA:	
		Shock room (*n* = 20/3.7%)	I (*n* = 18 / 3.3%)	
		OR (*n* = 2/0.4%)	II (*n* = 21 / 3.9%)	
			III (*n* = 222 / 40.7%)	NACA IV-VI: 38:09 min
			IV (*n* = 140 / 25.7%)	
			V (*n* = 100 / 18.3%)	
			VI (*n* = 10 / 1.8%)	
			VII (*n* = 33 / 6.1%)	
US pts. (n = 99)		Speciality:	∅ 4.5	40:26 min (σ 13:57)
		Medical (*n* = 55/55,6%)	(σ 1.2 / CI 4.25–4.74)	
		TS (*n* = 27/27.3%)	NACA:	
		NS (n = 3/3%)	I (n = 1/1.0%)	
		Uro (n = 2/2.0%)	II (*n* = 0)	
		VS (n = 1/1%)	III (*n* = 18/ 18.2%)	37:52 min After exclusion of longer patient transport because of ultrasound findings.
		Neuro (n = 1/1%)	IV (*n* = 35/ 35.4%)	
		Resuscitation with ROSC (n = 8/8.1%) showed above. (Resuscitations without ROSC are not assigned to any speciality *n* = 9/9.1%).	V (*n* = 28 / 28.3%)	
			VI (*n* = 8 / 8.1%)	
			VII (*n* = 9 / 9.1%)	
Trauma (n = 31)			∅ 4.0	40:35 min (σ 11:54)
			4.0 vs. 4.74: *P* < 0.001 (σ 0.93, CI 3.66–4.34)	
Non-trauma (n = 68)			∅ 4.74	40:21 min (σ 14:48)
			(σ 1.25, CI 4.43–5.04)	

*pts* patients, *CED* central emergency department, *US* ultrasound, *OR* operating room, *TS* trauma surgery, *NS* neurosurgery, *uro* urology, *VS* vascular surgery, *neuro* neurology, *ROSC* return of spontaneous circulation, *CI* confidence interval

Among the patients assessed with emergency ultrasound (*n* = 99), 90.0% were categorised as NACA III to VI. In the category NACA VII (death confirmed after unsuccessful resuscitation), 9 patients underwent emergency ultrasound scans during resuscitation. During the resuscitations which achieved return of spontaneous circulation (ROSC) (NACA VI), 8 of 10 patients had an ultrasound scan, while in 2 patients (allergic shock, STEMI) an ultrasound scan was not required as spontaneous circulation returned quickly.

Among the patients assessed with emergency ultrasound, the mean NACA score was significantly higher in the non-trauma patient group compared with the trauma patient group (Table 2).

The mean mission time in the emergency ultrasound group (40 min 26 s) was longer compared with the mean mission time in the total (patient population (34 min 12 s) (Table 2).

The mission time with and without ultrasound examination was analysed in each NACA score group (NACA III to NACA VI). It has been shown, that there is no significant difference between the mission times with or without ultrasound examination (NACA III: 41,1 min vs. 40,7 min, *p* = 0,822; NACA IV: 40,1 min vs. 36,04 min, *p* = 0,12; NACA V: 41,1 min vs. 40,8 min, *p* = 0,904; NACA VI: 60,9 min vs. 54,5 min, *p* = 0,551).

We also performed a regression analysis to identify the variables which affect the mission times. Two variables (NACA III to VI and ultrasonography) were tested to have the influence on the mission time. NACA changed the mission time significantly (*p* < 0,001) but not the ultrasonography (*p* = 0,123). In the NACA category I and II no missions with ultrasound examination were performed.

The prehospital emergency ultrasound was most often used in patients with dyspnoea prior to cardiac arrest as well as fall, followed by high-speed trauma, hypotension and polytrauma. All other indications occurred in less than 5% of cases (Table 3).

**Table 3:** Indications and procedures

	Indications:	1st priority	2nd priority	3rd priority
Trauma				
*n* = 31 / 31.3%	High-speed trauma *n* = 11 / 35.5%	FAST *n* = 11 (100%)	PLUS *n* = 10 / 90.9%	FEEL *n* = 3 / 27.3%
	Polytrauma *n* = 9 / 29.0%	FAST *n* = 9 / 100%	PLUS *n* = 9/ 100%	FEEL *n* = 6 / 66.7%
	Fall *n* = 8 / 25.8%	FAST *n* = 8 / 100%	PLUS *n* = 6 / 75.0%	FEEL *n* = 6 / 75.0%
	Resuscitation *n* = 2 / 6.5%	FAST *n* = 2/ 100%	PLUS *n* = 2/100%	FEEL *n* = 2 / 100%
	Chest trauma *n* = 1 / 3.2%	PLUS *n* = 1/ 100%	FEEL *n* = 0	FAST *n* = 1 / 100%
	Isol. abd. Trauma *n* = 0	FAST *n* = 0	FEEL *n* = 0	PLUS *n* = 0
Non-trauma				
*n* = 68 / 68.7%	Dyspnoea *n* = 38 /55.9%	PLUS *n* = 35/92.1% (6× PLUS alone =15.8%)	FEEL *n* = 29/76.3% (3× FEEL alone =7.9%)	
	Resuscitation *n* = 15 / 22.1%	FEEL *n* = 15/ 100%	PLUS n = 3 / 20.0%	FAST *n* = 1 / 6.7%
	Hypotension *n* = 8 / 11.8%	FEEL n = 8 / 100%	FAST n = 1 / 12.5%	PLUS *n* = 5 /62.5% all PLUS combined with FEEL
	Chest pain n = 4 / 5.9%	FEEL *n* = 3 / 75%	PLUS *n* = 2/50% (1× PLUS alone)	FAST *n* = 0
	Acute abdomen *n* = 2 / 2.9%	FAST *n* = 2 / 100%		
	Unclear symptoms *n* = 1 / 1.4%	FEEL *n* = 1 / 100%		

*FAST* Focused Assessment with Sonography for Trauma, *PLUS* Prehospital Lung Ultrasound, *FEEL* Focused Echocardiography in Emergency Life Support

The FAST, FEEL and PLUS ultrasound protocols were used in combination in the majority of examinations. A FAST emergency ultrasound examination was performed in all trauma patients, while the majority of patients with the most common principal symptom “dyspnoea” underwent a PLUS examination. FEEL examinations were performed in all patients after resuscitation, with the exception of 2 patients with fast ROSC.

Among the altogether 99 patients assessed with emergency ultrasound, the combinations of the various protocols were as follows: 36 patients (36.4%) underwent FEEL and PLUS examinations, 21 (21.2%) FEEL alone, 19 patients (19.2%) FAST, FEEL and PLUS examinations, 11 (11.1%) FAST and PLUS examinations, 6 (6.1%) PLUS alone and 5 (5%) FAST alone.

The following combinations were used in the trauma group and in the non-trauma group: In the trauma group with 31 patients, 17 (54.8%) patients underwent FAST, FEEL and PLUS examinations, 11(35.5%) FAST and PLUS examinations, and 3 (9.7%) FAST alone.

In the non-trauma group with 68 patients, 36 (52.9%) patients underwent FEEL and PLUS examinations, 21 (30.9%) FEEL alone, 6 (8.8%) PLUS alone, 2 (2.9%) FAST alone and 2 (2.9%) FAST, FEEL and PLUS examinations.

The mean ultrasound examination time with the FEEL protocol was 58 s, with FAST 1 min 2 s, with FAST and PLUS 1 min 14 s, with FEEL and PLUS 1 min 45 s, and with PLUS alone 33 s. The mean time for the complete ultrasound examination (FAST, FEEL and PLUS) was 2 min 27 s.

The examiners rated the imaging conditions with FAST examinations as “good” in 94.4% of cases (*n* = 34) and in the remaining cases (*n* = 2; 5.6%) as sufficient; in 1 case, placement of the transducer was complicated by a pelvic sling. With FEEL examinations (*n* = 77), examination quality was rated as sufficient in 71 (92.2%) cases and as poor in 6 (7.8%) cases. The subcostal (n = 34; 44.2%) and the apical (*n* = 22/28.6%) transducer placement locations were rated as the best positions for FEEL examinations. In 4 (5.2%) patients, the heart could not be visualised in an adequate time from any transducer position due to poor imaging conditions.

In the non-trauma patient group, prehospital echocardiography (FEEL) was performed in 50 cases. Amoung this group normal contractile function was found in 20 (40%) patients and impaired myocardial contractile function in 30 (60%) patients. While in hospital, 39 patients underwent follow-up echography; 34 pairs could be created which were analysed for impaired myocardial contractile function in both the prehospital and in-hospital examinations. Out of 34 pairs in the prehospital situation, normal left ventricular contractile function was found in 13 (38,2%) patients and impaired left ventricular contractile function in 21 (61,8%) patients. In-hospital echocardiography demonstrated good left ventricular contractile function in 11 (32,4%) patients and impaired contractile function in 17 (50%) patients (*n* = 34, sensitivity 89,4%; specificity 73,33%; positive/negative predictive value 0,809/0,846) The difference between the prehospital and in-hospital findings was not significant (*p* = 0.688, McNemar test for repeated categorical data of the same patient).

For the parameter “right ventricular stress”, the prehospital (ultrasound) examination was compared with imaging studies in the hospital (ultrasonography, CT scan); in 6 of 7 cases the prehospital diagnosis was confirmed (sensitivity 85,7%).

In 72 patients, a prehospital lung ultrasound (PLUS) was performed. Lung interstitial syndrome diagnosed with prehospital ultrasound scan was correlated with the findings of conventional chest x-rays in the hospital. In this subgroup there were 18 (25%) positive PLUS examination findings and 17(23,6%) positive chest x-ray findings (sensitivity 100%; specificity 96,15% positive/negative predictive value 1,0/0,96). Once again, the difference between the prehospital ultrasound findings and the findings of in-hospital investigations was not significant.

In 71 PLUS examinations, the diagnosis “ruling out pneumothorax” was confirmed (specificity 100%) by the examinations in the hospital.

Out of 33 FAST exams free fluid in the Koller pouch was shown in one patient; this finding was later confirmed in the hospital with ultrasonography and CT scan. In another patient, the presence of free fluid in the pouch of Douglas suspected during the prehospital ultrasound examination was not confirmed by CT scan and ultrasonography in the hospital.

In 2 cases, the bleeding (one from a perforated infrarenal abdominal aortic aneurysm into the retroperitoneal space and one mesenteric bleeding after a fall) could not be detected via the FAST I to IV transducer positions. Bleeding was diagnosed in the subxiphoid view when the transducer was tilted caudally; in one case already during the prehospital examination and in the other only after admission to hospital (specificity 97,1%).

Considering the combined prehospital findings (clinical examination, emergency ultrasound) the prehospital diagnosis “left ventricular decompensation” was confirmed in 89.5% of cases (19 positive prehospital findings vs. 17 positive in-hospital findings).

In 12 out of 99 cases (12.1%), the treating rescue physician arrived at the diagnosis “COPD exacerbation/pneumonia”; this diagnosis was confirmed in hospital in 11 patients (91.7%).

The correctness of the prehospital diagnosis in patients who underwent an ultrasound examination at the emergency site was confirmed in 90.8% of cases.

The emergency ultrasound findings triggered changes in altogether 66 invasive and non-invasive treatments in 49 (49.5%) of 99 patients. In the trauma patient group (*n* = 31), this was the case in 12 (38.7%) patients, while among non-trauma patients (*n* = 68) management was changed in 37 (54.4%) cases.

After ultrasound examination, patient transport destination, patient transport priority or monitoring requirements (e.g. patient does not need to be accompanied by a physician), changed in 33 of 99 cases (16 of 31 trauma cases; 17 of 68 non-trauma patients); the differences between the groups were statically significant (*p* = 0.009). For example, based on the prehospital ultrasound findings at the emergency site, ‘shock room’ management in the destination hospital (shock room alarm) was avoided in 8 cases, while 9 patients were transported to a more distant specialist hospital after ruling out free abdominal fluid, or in one case a stable patient with perisplenic free fluid was directly (not via the emergency rooms) transferred to the CT and from there to the operating room.

Altogether 17 resuscitations (8 x ROSC) were included in the study. At a time of the clinical decision to terminate the resuscitation and after ruling out other causes (cardiac tamponade, massive pulmonary embolism) of cardiovascular arrest, ultrasound imaging to confirm the absence of heart wall motion was performed.

## Discussion

### Key results

In our study a total of 99 (18.1%) emergency ultrasound examinations were performed during 546 callouts. The most frequent indications for the performance of prehospital emergency ultrasound were dyspnoea (*n* = 38; 38.4%), during cardiac arrest (*n* = 17/17.2%), fall (*n* = 12/12.1%) and high-speed trauma (*n* = 11/11.1%). In most cases, combinations of ultrasound examination protocols were applied (FAST/FEEL/PLUS).

Ultrasound-related changes in patient management occurred in 49.5% of patients. The emergency ultrasound findings were confirmed in 90.8% of cases in the hospital.

### Limitations

The aim of this study was to identify the range of emergency situations in which prehospital ultrasound was used while excluding “confounders” (except potential investigator subjectiveness). In prehospital emergency medicine, the ideal situation of a rescue team achieving expert standard in both emergency treatment and emergency ultrasound is not always given. The results of this study with a rate of emergency ultrasound examinations of approximately 18% cannot be applied to the standard operation of a rescue physician base, including our own.

Some limitations of the study refer to the methods. The ultrasound images at the site of emergency were not stored for a subsequent analysis. The findings of prehospital ultrasound examinations had to be compared with in-hospital results obtained not only by ultrasound, but also by different methods (CT-scans, x-rays) with different range of sensitivity and specificity, which may affect the diagnostic accuracy.

Finally the results might differ in a bigger population, where more patients with positive findings can be ruled in.

In the acute management of critically ill and injured patients, emergency ultrasonography is one of the established diagnostic modality in hospital care, both in the emergency department and on the wards. A number of important life-threatening conditions can—without delay—be detected by ultrasound examinations [1–3, 5, 6, 13, 14].

Besides the detection of free fluid, indicative of acute intraabdominal or intrathoracic bleeding, the lack of pleural sliding in patients with pneumothorax is another “guiding ultrasound finding” in trauma patients with impact on acute management [10].

In patients with “hypotension” as the principle symptom, the cause (pulmonary embolism, impaired contractile function, pericardial effusion, hypovolaemia) can be identified by ultrasound scanning and the further diagnostic workup and treatment can be started immediately with a high degree of confidence.

The same applies to “dyspnoea” as the principal symptom. Ultrasonography can help identifying many conditions in the differential diagnosis of dyspnoea (pulmonary embolism, heart failure, pericardial effusion, pneumonia, among others) [1, 2, 4, 5].

Ultrasound examinations might be as useful and beneficial for establishing the diagnosis in rescue medicine at the emergency site as they are in a hospital setting.

The percentage of emergency ultrasound examinations in this study (18,1%) exceeds the extent of prehospital ultrasound examinations reported in other and our previous studies (0.9 to 6.5%) by far [6, 15], but is in the same order of magnitude as the results of the study by Andersen et al. on the use of ultrasound examinations in a hospital setting [11]. In their study, every physician had a pocket-size ultrasound scanner readily available. During admission to hospital, ultrasound examinations were performed on 20.1% of patients (199 of 992 patients). The results of our study indicate that emergency ultrasound examinations can be performed as frequently at the emergency site in the prehospital situation as they are in the emergency department of a hospital.

The significantly higher mean NACA score in patients assessed with emergency ultrasound could be explained by the fact that in the most seriously ill or injured patients the most extensive diagnostic workup was undertaken. For example, ultrasound examinations were only performed at the emergency site on patients categorised as NACA III to VII with only one exception (Table 2).

Mean mission time increased with the severity of the condition (NACA score). Consequently, the mean mission time in the emergency ultrasound group (NACA 4.5) is longer compared with the mean mission time in the total (NACA 3.7) patient population (40 min 26 s and 34 min 12 s, respectively) (Table 2).

The ultrasound examination times were relatively short compared with other studies [6, 16]. To perform a FAST or FEEL examination required approximately 1 min, while a PLUS examination required approximately half a minute (FAST: 1 min 2 s, FEEL:58 s, PLUS:33 s). This is in line with the examination times reported by Busch [17], which can be regard as an “expert standard”. Studies with higher numbers of examiners (and cases), including our own studies, found longer ultrasound examination times [6, 11, 16]. However, during rescue physician missions the average 3-min emergency ultrasound examination time is not exceeded.

In our study the prehospital emergency ultrasound did not prolong the mission time. This is one of the basic findings as time consumption for the examination is considered to be one of the major concerns [8].

The use of emergency ultrasound in 15 (88.2%) of 17 cardiac arrest patients (NACA VI and VII) was considered almost “obligatory”. Only in two cases with resuscitation, the ultrasound examination recommended in the treatment algorithm was not performed due to the fast return of spontaneous circulation (1 case of anaphylaxis, 1 case of ST elevation myocardial infarction). The concept of ultrasonography with subxiphoid view during resuscitation (max. 10 s between two cardiac massage cycles) developed by Breitkreutz and Walcher [12, 18], among others, has thus become a standard procedure in our rescue physician missions. Certainly the clinical diagnostics and treatment stays prior to the use of technical equipment.

The choice of ultrasound protocol to be used for emergency ultrasound examinations in trauma patients (Table 3) is based on the seriousness of the risk associated with potential injuries [9]. By combining the standard examination according to the FAST protocol (detection of internal bleeding) with pleural and lung ultrasound (PLUS) and echocardiography (FEEL), important life-threatening conditions, such as pneumothorax and cardiac tamponade, can be ruled out [14]. Thus, in our study PLUS and/or FEEL examinations were performed in over 90% of trauma patients in addition to FAST examinations (Table 3).

In the non-trauma patients group, lung interstitial syndrome, impaired cardiac pump function and right ventricular stress play a prominent role. These conditions were detected in most cases using cardiac and chest ultrasonography according to the FEEL and FEEL+PLUS protocols [5, 19] (Table 3).

In the majority of patients, the prehospital ultrasound examination findings were confirmed by in-hospital investigations (sensitivity: impaired left ventricular contractile function, 89.4%; right ventricular stress, 85.7%; lung interstitial syndrome: 100%).

Prosen et al. and Laursen et al. showed in their studies a high sensitivity for the finding “lung interstitial syndrome” (100 and 94.4%. respectively) [5, 19].

According to our observations, lung ultrasound examinations to confirm or rule out interstitial fluid accumulation are performed rather infrequently. In a hospital setting, the most frequently used method to identify lung interstitial syndrome is conventional chest radiography.

We also found that not every patient underwent an ultrasound examination immediately after admission to hospital. For example, in case of cardiac recompensation (potentially already induced by prehospital treatment) it may not be possible to detect in the initial radiographs or CT scan the reduced cardiac pump function present in the acute phase.

The prehospital findings with regard to impaired left ventricular contractile function, right ventricular stress and lung interstitial syndrome were analysed in comparison with the in-hospital findings, using the McNemar test for repeated categorical data. The prehospital and in-hospital findings showed a high degree of agreement (*P* = 0.688 to 1.0).

On this stage it is important to mention that the diagnostic accuracy of ultrasound in detecting or ruling out pulmonary embolism is still a matter of research [20]. Decisions based on ultrasound findings can save lifes, but also worsen the outcome, if ultrasound is trusted too much. We have to be cautious about making too early decisions based only on ultrasound findings without considering alternative diagnostic pathways.

A “change in management” in response to the prehospital emergency ultrasound findings occurred in 49 (49.5%) of 99 patients. Neesse et al. [16] changed in patients diagnosed with acute coronary syndrome, heart failure and COPD the management in 25% of cases after prehospital ultrasound examinations of the lungs (FEEL, PLUS) and in 68% of patients the prehospital emergency ultrasound was rated as helpful.

In one third of patients, changes were made in response to prehospital ultrasound findings with regard to the destination and/or priority of patient transport or the monitoring requirements (e.g. rescue physician does not need to accompany patient during transport) or the procedures in the emergency department of the hospital (e.g. no shock room alarm).

It is an important aim of rescue service provision to emergency patients in rural areas to carefully use the resources both of the rescue service and the hospitals. If prehospital diagnosis helps to free medical staff, it improves the availability of the rescue physicians for other emergencies in the region.

In our study, the most common indication for prehospital emergency ultrasound was dyspnoea (38.4%). Since the cause of dyspnoea is often difficult to identify by physical examination alone (difficult differential diagnosis: cardiac decompensation vs. COPD/pneumonia) [5, 16, 19], and the seriousness of the risk is considered high in patients with this principal symptom, we suggest to consider performing a pleural and lung ultrasound in all patients presenting with this symptom. This applies to both the rescue physician mission and the management in the hospital.

However, diagnosis and treatment are not completed during the rescue physician mission but in hospital. In the prehospital situation, emergency ultrasound should be used to answer questions that improve the quality of care and the choice of the patient transport destination. Fast patient transport to a hospital (Table 2) where the required diagnostic and therapeutic procedures (surgery, coronary or cerebral artery interventions, etc.) can be performed is another priority [21]. Prehospital emergency ultrasound should not be performed “because one knows how to do it”; it should be used because it provides a benefit to the patient at the emergency site.

The reason for the slow introduction of prehospital emergency ultrasound is the significant time and logistic effort required to train all rescue physicians at a rescue service base in emergency ultrasound to a level of proficiency where meaningful findings are obtained in less than 60 s or 120 s (FAST and FEEL, respectively). After in-depth training (e.g. based on Anaesthesia-Focussed Sonography (AFS) modules, Focused Abdominal Sonography for Trauma/Focused Echocardiography in Emergency Life Support (FAST/FEEL) courses) [7, 12, 13], maintaining and adding to these skills requires regular training sessions for all emergency physicians (at our emergency physician base at least 30 min per week).

## Conclusions

Emergency ultrasound was frequently performed in the prehospital setting and the physicians seemed to combine protocols based on the individual patient. The examinations revealed significant pathology that changed patient-management and prehospital ultrasound findings correlated well with in-hospital diagnostic findings.

## Abbreviations

AFS: Anaesthesia-Focussed Sonography
BMI: Body mass index
BP: Blood pressure
CED: Central emergency department
CI: Confidence interval
COPD: Chronic obstructive pulmonary disease
CT: Computed tomography
ECG: Electrocardiography
ERC: European Resuscitation Council
FAST: Focused Assessment with Sonography for Trauma
FEEL: Focused Echocardiography in Emergency Life Support
HR: Heart rate
ICU: Intensive care unit
NACA: National Advisory Committee for Aeronautics scoring system of the severity in cases of medical emergencies
Neuro: Eurology
NS: Neurosurgery
nTr: Non-trauma
OR: Operating room
PLUS: Prehospital Lung Ultrasound.
Pts: Patients
ROSC: Return of spontaneous circulation
STEMI: ST-segment elevation myocardial infarction
Tr: Trauma
TS: Trauma surgery
Uro: Urology
US: Ultrasound
VS: Vascular surgery
σ: Standard deviation
